# The Influence of Pre-Competitive Anxiety and Self-Confidence on Dancesport Performance

**DOI:** 10.3390/sports12110308

**Published:** 2024-11-13

**Authors:** Sara Aliberti, Gaetano Raiola, Francesca D’Elia, Domenico Cherubini

**Affiliations:** 1 Facultad de Deporte, UCAM Universidad Católica de Murcia, 30107 Guadalupe, Spain; dcherubini@ucam.edu; 2 Research Center of Physical Education and Exercise, University of Pegaso, 80143 Napoli, Italy; gaetano.raiola@unipegaso.it; 3 Department of Human, Philosophical, and Educational Sciences, University of Salerno, 84084 Fisciano, Italy; fdelia@unisa.it

**Keywords:** anxiety, assessment, self-confidence, competition, dancers

## Abstract

Competitive dancesport (DS) performance is a multifactorial phenomenon influenced by physical and mental factors. The emotions experienced by athletes in competition are strongly linked to their sports performance. However, to our knowledge, no studies have investigated the direct relationship between performance and emotional states in DS. Consequently, the aims were four: (I) to investigate the influence of anxiety and self-confidence on DS performance; (II) to examine the influence of years of experience, prior victories, and perceived preparedness on performance outcomes; (III) to identify the optimal emotional state levels for peak performance; (IV) to investigate differences between different athletes’ levels and class. The participants were 71 Italian DS athletes divided into three groups (22 B-class, 25 C-class, 24 D-class). Before competition, they supplied demographic information about their gender, years of experience, perceived preparedness, previous winnings in the current class, followed by the completion of the Italian version of Revised Competitive State Anxiety Inventory–2 (CSAI-2R). To assess the athletes’ performance, the final classification of the competition was taken into consideration. The results showed that both overall and relative variables from the CSAI-2R significantly predicted performance outcomes (*p* < 0.05), although somatic anxiety did so to a lesser extent. Significant differences emerged between athletes of different classes in terms of years of experience (*p* = 0.000), perceived preparedness (*p* = 0.000), cognitive anxiety (*p* = 0.000) and self-confidence (*p* = 0.000). The optimal levels for good performance were cognitive anxiety (11.61 ± 2.27), somatic anxiety (15.77 ± 1.72) and self-confidence (15.12 ± 2.56). The findings of this study provide valuable insights into the multifactorial nature of competitive DS performance, particularly highlighting the significant role of emotional states such as anxiety and self-confidence, as well as other variables such as class, level, years of experience and perceived preparedness.

## 1. Introduction

The emphasis our society has placed on competition has made sport a significant field for psychological studies [1,2]. Competitive dancesport (DS) is a multifactorial phenomenon influenced by several factors [3] that can provoke both physical and mental responses. Managing emotional states represents one of the main challenges athletes have to face [4]. According to Gallwey [5], performance could be considered as the result of one’s potential less the interference from factors such as anxiety, stress, or lack of self-confidence [6]; to achieve peak performance, athletes need to minimize the interference. Working to effectively manage these interferences could allow athletes to fully express their potential during competition. According to a recent literature review conducted by Aliberti et al. [7], anxiety was the most frequent emotional state among DS athletes, followed by stress, arousal, and self-confidence. Sports psychologists differentiate trait anxiety, which refers to a more stable aspect of personality and state anxiety, related to feelings linked to a particular situation [8]. State anxiety is further classified as somatic anxiety and cognitive anxiety. Cognitive anxiety refers to symptoms such as confusion, indecision, negative thoughts, irritability, fear, sense of failure, dissatisfaction, and avoidance. Somatic anxiety refers to symptoms including increased blood pressure and respiration rate, sweating, nausea, muscular tension, trembling, tightness in different body parts, clammy hands, dry mouth, need to urinate, loss of appetite, and diarrhea [9]. Accepting anxiety symptoms as a natural part of the competitive experience is fundamental so that they can contribute to facilitating performance. Instead of fighting or trying to eliminate them, it is important for athletes to learn to manage and channel anxiety positively [10].

Different theories have analyzed the relationship between anxiety and performance, with the most accredited being the Inverted U theory, the multidimensional theory, and the Individual Zone of Functioning (IZOF) theory. The Inverted U theory posited the existence of an inverted U-shaped relationship between arousal and performance, where increasing arousal improved performance up to the peak of the U, after which performance declined [11]. Martens et al. [12] proposed a theory stating a linear negative relationship between cognitive anxiety and performance, an inverted U-shaped relationship between somatic anxiety and performance, and a decrease in somatic anxiety after the start of the performance, unlike cognitive anxiety, which remains high when the athlete exhibits low self-confidence. One of the most recent theories is the IZOF, which emphasizes the uniqueness of each athlete in responding to competition anxiety, identifying peak performance within an individual’s optimal functioning zone, characterized by the appropriate levels of arousal or anxiety. However, how these variables influence DS performance remains largely unexplored [7]. Specifically, identifying the optimal ranges of anxiety and self-confidence for achieving good performance would be beneficial.

The motivational aspect and personal experience are also not to be overlooked, as they could influence an athlete’s perception of security and competence, thus affecting performance outcomes [13] (drive theory). A previous victory often influences performance in subsequent competitions, especially when the competitors in the next competition are the same [14]. Local competitions typically involve local athletes who are already familiar with each other. This familiarity creates a psychological backdrop that significantly impacts the athlete’s mindset and approach to the competition. When athletes compete against familiar competitors, the psychological effects of past performances become more pronounced. A previous victory can boost an athlete’s confidence, creating a positive feedback loop that enhances future performance [15]. Conversely, a previous defeat can lead to heightened anxiety and self-doubt, particularly if the athlete perceives the competition as a recurring challenge against the same competitors.

The perception of competence is another variable that influences the outcome of sports performance through its impact on motivation, resilience, self-esteem, and approach to challenges. Athletes who perceive themselves as competent are more likely to fully engage in sports activities, face challenges with determination, and maintain confidence in their abilities in competition [16]. Finally, years of experience are another useful variable, as they influence the outcome of sports performance through the improvement of technical and tactical skills, a greater understanding of the competitive context, better stress and pressure management, and increased confidence in their abilities [17].

The emotions experienced by athletes in competition are strongly linked to their sports performance. The hours before the competition are characterized by a gradual increase in both positive and negative emotions. Their control helps athletes to optimize their performance, as they need to interpret competition choreographies in addition to executing the technical aspects.

To our knowledge, no studies have investigated the direct relationship between performance and emotional states in DS. A recent systematic review [7] revealed only four studies that addressed the topic of emotions in DS, without measuring sports performance. In particular, the study by Andreeva and Karanauskienė [6] considered a qualitative analysis that showed that athletes perceived that emotions could influence performance either positively or negatively. Ermolaeva [10] measured the level of anxiety in DS athletes through the Spielbierg questionnaire, showing a high level of state anxiety before competition. Čačković et al. [4], through theoretical analysis, showed that in DS competitions athletes face many stressors, emphasizing the need to improve their psychological preparation. Finally, Pakulanon and Poomsalood [3], through the Revised Competitive State Anxiety Inventory–2 (CSAI-2R), analyzed the level of anxiety and self-esteem before and after competition, highlighting the need to manage anxiety at least one week before the competition. The CSAI-2R has been widely studied for its role in measuring pre-competition anxiety and self-confidence. Several meta-analyses, such as that by Craft et al. [18], established that cognitive and somatic anxiety typically demonstrate a negative correlation with performance across various sports domains, while self-confidence shows a positive association. These analyses provide a benchmark for understanding how these psychological states interact with performance. Incorporating these perspectives allows us to situate our investigation within the broader context of sports psychology while highlighting the unique aspects specific to dance, such as the combination of artistic expression and technical precision. Unlike many sports that emphasize raw physical output, dance combines athletic skill with artistry. This dual focus intensifies the psychological pressure, as dancers must not only execute technically demanding movements but also convey emotion and character, often in synchrony with music. The CSAI-2R’s ability to capture both cognitive concerns and somatic symptoms makes it a suitable measure for exploring how these factors impact dancers’ competitive performance.

Consequently, the aims were four: (I) to investigate the influence of anxiety and self-confidence on DS performance; (II) to examine the influence of years of experience, prior victories, and perceived preparedness on performance outcomes; (III) to identify the optimal emotional state levels for peak performance; (IV) to investigate the differences between different athletes’ level and class. The hypotheses were: (H_1_) pre-competitive anxiety and self-confidence had a significant influence on DS athletes’ performance; (H_2_) years of experience in the DS field and perceived preparedness significantly influenced the performance outcomes, mediating the effect of pre-competitive emotional states; (H_3_) there were optimal levels of anxiety and self-confidence that could maximize the performance of DS athletes; (H_4_) the levels of anxiety and self-confidence varied according to the athlete’s level (high and low) and class.

## 2. Materials and Methods

### 2.1. Design and Participants

The design of the study was observational. Initially, participants were 71 Italian DS athletes recruited through convenience sampling, divided into three groups:

- Twenty-two athletes of medium level (B-class)
- Twenty-five athletes of low level (C-class)
- Twenty-four athletes of promotional/starting level (D-class)

However, after a preliminary analysis of the data, 9 participants were identified and excluded, as they were identified as outliers, according to Cook’s test, regarding the CSAI-2R final score. Therefore, the final number of analyzed participants was 62 (age, 23 ± 3.2; 100% female), divided into three groups: 20 B-class athletes, 24 C-class athletes, 18 D-class athletes. Inclusion criteria were Italian DS athletes from solo discipline of different classes of the over-17 category. Informed consent was obtained. The study was conducted according to the guidelines of the Declaration of Helsinki. The study was approved by the Research Ethics Committee of the Catholic University of Murcia Code CE072315 (21 July 2023).

### 2.2. Data Collection

During a local competition day, participants completed a questionnaire in paper form administered during a local competition day divided into two sections: the first aimed to find out the demographic information of the participants, including their experience in DS field, while the second section included the Italian version of the Revised Competitive State Anxiety Inventory–2 (CSAI-2R).

#### 2.2.1. Demographic Characteristics and Personal Experience

This section included the following questions: (1) What is your gender? (2) What is your age? (3) What are your years of DS experience? (4) How do you perceive yourself in preparedness to compete, from 1 (poor) to 10 (high)? (5) Have you already won in your current class?

#### 2.2.2. CSAI-2R

The CSAI-2R, developed by Martens et al. [12], revised by Cox et al. [19] and translated into Italian by Martinengo et al. [20], was used to assess the level of anxiety and self-confidence perceived before competition. It was composed of 17 items that measured cognitive, somatic anxiety, and self-confidence. Participants rated each item on a 4-point Likert scale ranging from one (not at all), two (somewhat), three (moderately so), to four (very much so). A low score indicated the athlete had low anxiety (high self-confidence), while a high score indicated high anxiety. Scores ranged from 10 to 40 for each subscale. The questionnaire is a valid and reliable tool for scholars and practitioners in the field of sport and applied social psychology [20], with a Cronbach’s alpha coefficient over 0.8.

The questionnaire administration was in paper form, taken 30–50 min before their performance. Self-report measures have limitations, both because their interpretation is subjective and because they can disrupt the pre-race condition; however, several researchers have proposed some strategies to overcome them, such as modifying the timing of questionnaire administration. Administering the questionnaire too early, such as 15–30 min before competition, may distract athletes from performance. Furthermore, it would not guarantee the veracity of the answers as the athletes in this time slot are not focused on answering the questionnaire as they are most likely already cognitively immersed in preparation [21]. According to the scientific literature, the ideal time was 1 h before competition for accurate results [18], an example followed by Marwat et al. [22], Hussain et al. [23] and others. This time frame has been consistently used in the literature and is considered acceptable as it does not interfere with preparation routines [24]. Although its primary use has been in traditional sports, its applicability extends to any domain involving performance under competitive conditions. The CSAI-2’s focus on cognitive and somatic responses aligns well with the psychological profile of dancers who face unique challenges, such as performing complex routines in front of judges and audiences while under time constraints. The mental and physical symptoms measured by the CSAI-2R are particularly pertinent given that dancers must balance anxiety management with maintaining poise and expression.

#### 2.2.3. Performance Assessment

Performance was measured using the athletes’ final competition ranking on the day of assessment, representing their outcome in that specific event. This approach helps establish a direct relationship between pre-competitive psychological states and immediate competitive results rather than a general assessment of their skill or competitive history. The official database of the organizing body was used to view the ranking. A value of one (1) indicated that the athlete ranked higher than the others, so a lower value corresponded to high performance.

### 2.3. Statistical Analysis

Descriptive statistics were used to summarize the characteristics of a data set. Reliability of the CSAI-2R dimensions was examined through the calculation of Cronbach’s alpha. A score of 0.7 or above was considered good, indicating that the scale was internally consistent. A score of 0.5 or below meant that the questions needed to be revised or replaced, and in some cases, that the scale needed to be redesigned [25]. Pearson’s correlation (r) was performed to measure the strength and the direction between two variables. The following criteria were adopted to interpret the magnitude of correlations between measurement variables: 0.1–0.3 (small), 0.3–0.5 (moderate), 0.5–0.7 (large), 0.7–0.9 (very large), and 0.9–1.0 (almost perfect) [26]. Linear and multiple regression were used to predict the performance ranking (dependent variable) based on the value of cognitive anxiety, somatic anxiety, and self-confidence (independent). One-way Anova was used to analyze differences in terms of years of experience, perceived preparedness and CSAI-2R subscales among athletes of different levels (D, C, B). Chi Square analysis was performed to analyze differences in terms of previous winning among athletes of different levels. Mancova was performed to test the influence of years of experience and perceived preparedness (from 1 to 10) on performance (ranking) and CSAI-2R using Class (D, C, B) as fixed factors. Additionally, as suggested by Fortes et al. [27], participants were divided into high performance (HP) and low performance (LP) groups based on their ranking positions. This division was made using the 50th percentile of the ranking distribution. The median of this distribution was used as the cutoff point. Athletes with a ranking position above the median were classified as LP athletes, while those below the median were classified as HP athletes. To examine the differences in CSAI-2R among athletes of different levels (D, C, B) and with different performance levels (HP, LP), a two-way ANOVA was conducted to assess the effects of class and performance level, as well as the interaction between these factors. Significance was set at *p* ≤ 0.05. Data analyses were performed using the Statistical Package for Social Science software (IBM SPSS Statistics for Windows, version 25.0, IBM, SPSS Inc., Armonk, NY, USA).

## 3. Results

The assumptions of normality, linearity, multicollinearity, and homogeneity of variances were not violated. The normality test result was not statistically significant (*p*> 0.05). Similarly, the deviation from linearity was not statistically significant (*p*> 0.05). However, the Cook’s distance test identified some outliers, reducing the sample size from 71 to 62 athletes, comprising 18 athletes from class D, 24 athletes from class C, and 20 athletes from class B.

- What are the relationships between performance, anxiety, self-confidence, years of experience and perceived preparedness?

Significant relationships emerged from Pearson’s correlation. A detailed description is shown in Table 1.

- How do CSAI-2R variables affect performance rank?

The level of self-confidence (independent variable) explained 56% of the variability in the performance rank, F(1, 60) = 77.57, *p* < 0.001. The level of cognitive anxiety explained 55% of the variability in the performance rank level, F(1, 60) = 73.43, *p* < 0.001. The level of somatic anxiety explained 32% of the variability in the level of performance rank, F(1, 60) = 29.35, *p* < 0.001. From multiple regression analysis, CSAI-2R scores explained 67% of the variability in the performance rank level, F(58, 3) = 39.48, *p* < 0.001. A detailed description is shown in Table 2.

- What are the differences between different level athletes?

The internal consistency of the CSAI-2R dimensions was calculated using Cronbach’s alpha, yielding the following results: cognitive anxiety = 0.8 (excellent), somatic anxiety = 0.6 (acceptable), and self-confidence = 0.9 (excellent). From the one-way Anova, significant differences emerged between athletes of different levels in terms of years of experience F(2, 68) = 94.15, *p* = 0.00, perceived preparedness F(2, 68) = 7.16, *p* = 0.00, cognitive anxiety F(2, 68) = 4.41, *p* = 0.000 and self-confidence F(2, 68) = 10.97, *p* = 0.00. From the Chi Square analysis, no differences emerged in terms of previous winning χ2 ([1], N = [71]) = [2.20], *p* = [0.325]. A detailed description is shown in Table 3.

Note. M = mean, SD = standard deviation, percentages indicate the proportion of participants reporting previous wins; N/A, not applicable.

- How do years of experience, perception of preparedness, and class affect CSAI-2R and performance?

From the multivariate analysis, years of experience in competitive dancing (*p* = 0.008), perception of preparedness (*p* = 0.008), and athlete class (*p* = 0.001), all test values were significant, indicating that these variables had a significant combined effect on cognitive anxiety, somatic anxiety, self-confidence, and final score. Years of experience had a significant impact on cognitive anxiety, self-confidence, and final score, but not on somatic anxiety. Perception of preparedness had a significant impact on all variables. A detailed description is shown in Table 4.

- What is the ideal level for best performance?

The median (50th percentile) for the performance rank variable was calculated for each class, revealing that for class D, the performance rank median is 9.50, for class C it is 11.33, and for class B it is 10.37. Subsequently, athletes from the three classes were divided into HP (high performance) athletes and LP (low performance) athletes. A detailed description is provided in Table 5.

**Figure 1:**
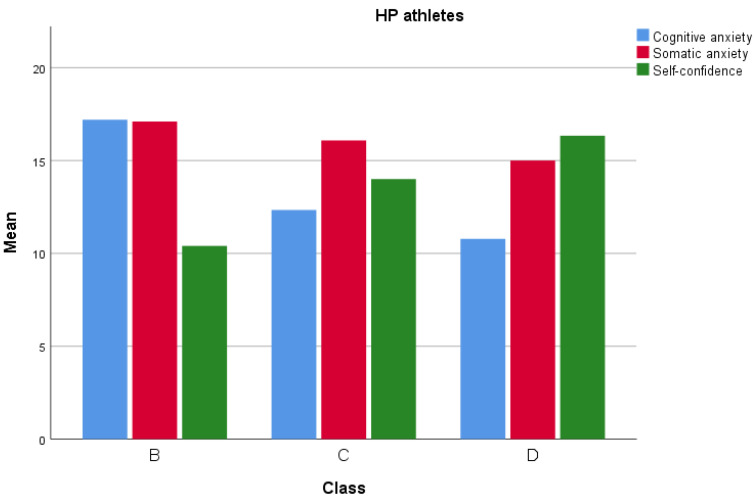
Best levels in HL athletes.

**Figure 2:**
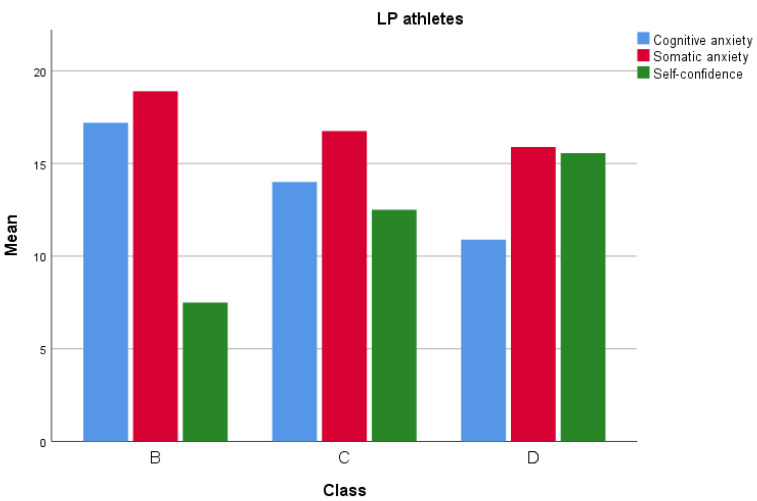
Worst level in LP athletes.

Figure 1 and Figure 2 represent the ideal levels of somatic anxiety, cognitive anxiety, and self-confidence in athletes of different levels, followed by the worst levels.

- What are the differences in CSAI-2R between athletes of different levels (D, C, B) and with different performance levels (HP, LP)?

From the two-way Anova, the performance level variable had a significant effect on self-confidence, as did the class variable and the interaction between class * performance level. From the post hoc Bonferroni, significant differences emerged between B–C and B–D (*p* = 0.00). The performance level variable had a significant effect on somatic anxiety, but the class variable did not have a significant effect. Additionally, there was no evidence of a significant interaction between class * performance level on somatic anxiety (*p* > 0.05). The performance level variable had a significant effect on cognitive anxiety, as did the class variable and the interaction between class * performance level. From Bonferroni, a significant difference emerged between B and C classes (*p* = 0.00). A detailed description is shown in Table 6.

## 4. Discussion

The results of this study confirmed the importance of pre-competitive anxiety and self-confidence in the performance of DS athletes, supporting hypothesis H_1_, which stated that pre-competitive anxiety and self-confidence significantly influenced DS athletes’ performance. Specifically, the findings showed that high levels of cognitive and somatic anxiety were correlated with poorer performance and reduced self-confidence, while greater self-confidence was positively correlated with better performance. This aligns with multidimensional anxiety theory, which suggests the stronger impact of cognitive anxiety on performance than somatic anxiety [28,29]. Regression analysis further demonstrated that cognitive anxiety, somatic anxiety, and self-confidence could influence ranking scores in DS. The regression model for self-confidence indicated a significant role in predicting DS performance. An increase in self-confidence was associated with a decrease in ranking positions, suggesting that athletes with higher self-confidence tended to achieve better results. In different sports, self-confidence has been one of the most significant predictors of performance [30,31]. Similarly, the regression model for cognitive anxiety showed a strong relationship with DS performance, where an increase in cognitive anxiety was associated with poorer performance, as supported by other studies [32,33]. By contrast, the regression model for somatic anxiety demonstrated a significant but weaker relationship compared to cognitive anxiety and self-confidence, with an increase in somatic anxiety slightly deteriorating the performance, consistent with other findings that indicate somatic anxiety has a significant impact on performance [34,35].

The data for hypothesis H_2_ also indicated that experience and perceived preparedness significantly affected performance outcomes, mediating the effect of pre-competitive emotional states. Athletes with greater experience reported lower levels of cognitive anxiety and higher self-confidence, suggesting that emotional management improves with experience. Additionally, a positive perception of one’s preparedness was associated with lower anxiety and elevated self-confidence, supporting focus during competition. These results align with the competence perception theory, which suggests that confidence in one’s abilities promotes more effective anxiety management and better performance [36,37]. Somatic anxiety appeared less influenced by experience, indicating that the physiological stress response may be more resistant to the effects of time and practice. Class membership significantly affected cognitive anxiety and final scores but not somatic anxiety or self-confidence, meaning somatic anxiety and self-confidence did not vary by athlete class.

Dividing the sample into two groups revealed significant differences between HP and LP athletes in cognitive anxiety, somatic anxiety, and self-confidence. Hypothesis H_3_ was partially confirmed by the analysis of optimal levels of anxiety and self-confidence for maximizing performance. Data showed that HP athletes had significantly lower levels of cognitive anxiety (M = 11.61, SD = 2.27) compared to LP athletes (M = 15.96, SD = 2.88), suggesting that top-ranking athletes experienced lower cognitive anxiety. High cognitive anxiety can interfere with concentration and decision-making, whereas lower levels of cognitive anxiety allow athletes to maintain mental calm and focus. Regarding somatic anxiety, HP athletes exhibited slightly lower levels (M = 15.77, SD = 1.72) than LP athletes (M = 17.51, SD = 1.94). Somatic anxiety levels did not vary by class within the HP and LP groups, indicating that regardless of class, athletes who keep somatic anxiety at moderate levels tend to perform better. HP athletes also had significantly higher self-confidence (M = 15.12, SD = 2.56) than LP athletes (M = 10.16, SD = 3.27). In summary, optimal performance is associated with moderate somatic anxiety, moderate self-confidence, and lower cognitive anxiety. This supports the IZOF theory, which proposes an “optimal functioning range” for each athlete, with moderate levels of anxiety and self-confidence enabling peak performance [38].

Concerning hypothesis H_4_, comparisons between athletes of different classes showed significant differences in years of experience, perceived preparedness, cognitive anxiety, and self-confidence. Specifically, B-class athletes had more years of experience than C-class and D-class athletes, while C-class athletes had more experience than D-class athletes, suggesting a gradual increase in experience with progression through classes. Regarding perceived preparedness, no substantial differences were found between C-class and B-class athletes or between D-class and C-class athletes, but a notable difference emerged between D-class and B-class athletes, with D-class athletes perceiving themselves as less prepared than their more experienced counterparts. Regarding levels of cognitive anxiety, C-class athletes experienced higher levels compared to B-class, followed by D-class, though the difference was not significant. B-class athletes showed lower levels of cognitive anxiety, possibly due to the more established environment, where athletes had demonstrated competitive ability. Finally, in terms of self-confidence, B-class athletes exhibited higher self-confidence than D-class athletes. Accumulated experience in the B-class likely contributed to more developed skills, opportunities to overcome challenges, and positive results [39]. Somatic anxiety levels, however, did not appear to vary by class, suggesting that somatic anxiety may be a common physiological response among athletes regardless of competitive level.

The multivariate analysis confirmed the significant influence of experience, perceived preparedness, and class on athletes’ emotional states and performance. Experience contributed to higher self-confidence and reduced cognitive anxiety, while somatic anxiety appeared less affected by experience, suggesting that the physiological stress response might be less sensitive to time and practice [37,40]. Perceived preparedness also impacted all the emotional states analyzed, underscoring the importance of a positive perception of one’s preparation for reducing anxiety and boosting self-confidence.

The study underscored the critical roles of pre-competitive anxiety and self-confidence in the performance of DS athletes. First, our findings demonstrated that high cognitive anxiety was correlated with poorer performance, aligning with the multidimensional anxiety theory [12]. Regression analysis further highlighted self-confidence as a key predictor of performance, consistent with Vealey’s [41] assertion regarding its importance in athletic success. Greater experience and perceived preparedness contributed to enhanced self-confidence and reduced cognitive anxiety, reflecting findings by Mellalieu et al. [42], suggesting that effective emotional management improved with time and experience. HP athletes showed lower cognitive anxiety, facilitating concentration and decision-making, an observation supported by research on optimal anxiety levels for improved performance. Interestingly, while somatic anxiety levels did not differ significantly by athlete class, maintaining moderate levels appeared beneficial for performance. Class-related variations in experience and confidence echo the literature linking accumulated experience to improved performance. These findings highlight the importance of developing targeted interventions to improve cognitive and somatic anxiety management and increase self-confidence in DS athletes, enabling optimal pre-competitive emotional management for maximizing performance. The strength of this study lies in its originality, being one of the first to explore the relationship between anxiety and performance in competitive DS. Furthermore, the study demonstrates the usefulness of the CSAI-2R in capturing the multifaceted nature of performance anxiety in competitive DS. This highlights its applicability beyond traditional sport contexts, as it effectively measures both cognitive and somatic aspects of anxiety, which are critical given the dual demands of technical execution and artistic expression inherent in dance.

## 5. Conclusions

This study confirmed the importance of pre-competitive emotional states, particularly anxiety and self-confidence, in influencing athletic performance in DS athletes. Regarding the hypotheses, H_1_ was confirmed, as both cognitive and somatic anxiety showed a negative impact on performance, with higher anxiety levels associated with lower ranking scores. Conversely, self-confidence had a positive influence, with higher self-confidence correlating with better performance. H_2_ was supported by findings that indicated years of experience and perceived preparedness significantly influenced performance outcomes by mediating the effects of pre-competitive emotional states. Experienced athletes and those who felt well-prepared showed lower levels of cognitive anxiety and higher self-confidence, facilitating better focus and resilience during competition. H_3_ was partially confirmed, suggesting optimal levels of somatic and cognitive anxiety as well as self-confidence for peak performance. HP athletes had moderate levels of somatic anxiety, lower cognitive anxiety, and higher self-confidence than LP athletes. H_4_ was confirmed by identifying differences between the athlete classes, where B-class athletes demonstrated greater experience, lower cognitive anxiety, and higher self-confidence compared to the lower classes. These findings underscore the need for targeted interventions to help DS athletes manage cognitive and somatic anxiety effectively while enhancing self-confidence. Practical applications include psychological training to foster emotional control and pre-competitive preparation, which may contribute to maximizing performance, particularly at advanced competitive levels. The limitations of this study include the sample size and its composition, which were limited to female Italian athletes in specific DS categories, potentially affecting generalizability to broader or more diverse athlete populations. Expanding the sample in future studies could enhance representativeness and enable further cross-group comparisons, particularly including athletes from the higher A-class category. Moreover, repeating assessments in varied pre-competitive contexts would provide a clearer picture of emotional stability across competitive events and allow for a distinction between trait anxiety and situational anxiety in DS. Overall, this study contributes to the emerging understanding of the emotional factors impacting DS performance and offers insights for practical application in sports psychology, reinforcing the value of emotional training in enhancing athlete resilience and performance outcomes.

## Figures and Tables

**Table 1:** Correlation analysis.

Correlations	1	2	3	4	5	6
**Performance ranking**	1					
**Years of experience**	−0.252 *	1				
**Preparedness perception**	−0.442 **	0.313 *	1			
**Cognitive anxiety**	0.742 **	−0.399 **	−0.362 **	1		
**Somatic anxiety**	0.573 **	−0.013	−0.390 **	0.460 **	1	
**Self-confidence**	−0.751 **	0.520 **	0.555 **	−0.768 **	−0.482 **	1

* Correlation is significant at 0.05 level (two-tailed); ** Correlation is significant at 0.01 level (two-tailed).

**Table 2:** Multiple regression analysis for CSAI-2R variables predicting performance rank.

Variable	B	SE	β	t	*p*	95% CI
**Constant**	−2.284	6.880		−0.332	0.741	[−16.055, 11.488]
**Cognitive anxiety**	0.652	0.221	0.351	2.947	0.005	[0.209, 1.096]
**Somatic anxiety**	0.727	0.270	0.234	2.689	0.009	[0.186, 1.268]
**Self-confidence**	−0.603	0.197	−0.369	−3.055	0.003	[−0.998, −0.208]

Note. R^2^ = 0.671, adjusted R^2^ = 0.654, F(3, 58) = 39.489, *p* < 0.001, std. error of estimate = 3.69746.

**Table 3:** Descriptive statistics for years of experience, frequency of training, perceived preparedness, previous winning, CSAI-2R and post hoc comparisons.

Item	D-Class (M, SD)	C-Class (M, SD)	B-Class (M, SD)	*p*	Bonferroni Correction
Years of experience	3.00 (1.41)	4.33 (1.55)	9.15 (2.01)	<0.001	D vs. C: 0.040; D vs. B: < 0.001
Perceived preparedness	6.27 (1.22)	6.83 (0.91)	7.20 (1.15)	0.039	D vs. B: 0.035
Previous winning (%)	Yes (44.4)	Yes (50.0)	Yes (55.0)	0.810	N/A
	No (55.6)	No (50.0)	No (45.0)		
Somatic anxiety	16.27 (2.05)	16.66 (1.92)	16.95 (2.16)	0.600	N/A
Cognitive anxiety	13.88 (3.84)	15.08 (2.50)	12.15 (3.31)	0.014	B vs. C: 0.011
Self-confidence	10.77 (3.50)	12.29 (3.16)	14.75 (4.02)	0.004	B vs. D: 0.003
Performance rank	10.68 (6.47)	11.85 (6.73)	10.85 (5.80)	0.808	N/A

**Table 4:** Multivariate tests.

Effect	Value	F	Hypothesis df	Error df	Sig.
Intercept	0.147	78.403	4	54	<0.001
Years of experience in DS	0.777	3.879	4	54	0.008
**Preparedness perception**	0.721	5.223	4	54	0.001
**Class**	0.57	4.376	8	108	<0.001

Note. Design: intercept + years of experience + perceived preparedness + class.

**Table 5:** Descriptive statistics for self-confidence, cognitive anxiety, and somatic anxiety by group.

	B		C		D	
	HP	LP	HP	LP	HP	LP
Self-confidence						
M (SD)	17.90 (1.37)	11.60 (3.20)	14.42 (1.38)	10.17 (3.04)	13.00 (2.12)	8.56 (3.24)
Cognitive anxiety						
M (SD)	9.90 (1.91)	14.40 (2.88)	13.08 (0.79)	17.08 (1.93)	11.56 (2.74)	16.22 (3.42)
Somatic anxiety						
M (SD)	15.90 (2.08)	18.00 (1.76)	16.17 (1.03)	17.17 (2.48)	15.11 (2.03)	17.44 (1.33)

**Table 6:** Two-way ANOVA results for somatic anxiety, self-confidence, and cognitive anxiety.

Dependent Variable	Source	Sum of Squares	df	Mean Square	F	Sig.	Bonferroni
**Somatic Anxiety**	Correct model	56.849	5	11.370	3.293	0.011	-
	Class	4.299	2	2.150	0.623	0.540	-
	Performance level	50.130	1	50.130	14.520	0.000	HP < LP
	Class * performance	5.518	2	2.759	0.799	0.455	-
**Self-Confidence**	Correct model	154.374	2	77.187	12.276	0.000	B > C, B > D
	Performance level	381.793	1	381.793	60.722	0.000	HP > LP
	Class * performance	13.198	2	6.599	1.050	0.357	-
**Cognitive Anxiety**	Correct model	94.113	2	47.057	8.531	0.001	B < C
	Performance level	294.387	1	294.387	53.367	0.000	HP < LP
	Class * performance	1.298	2	0.649	0.118	0.889	-

## Data Availability

The raw data supporting the conclusions of this article will be made available by the authors on request.
